# Layer-Specific Architecture and Nerve Innervation of the Popliteus Muscle: Neuroanatomical Basis for Precision-Guided Interventions for the Knee Joint

**DOI:** 10.3390/diagnostics16060834

**Published:** 2026-03-11

**Authors:** Soo-Jung Kim, Ji-Hyun Lee, In-Seung Yeo

**Affiliations:** 1 Department of Anatomy and Cell Biology, Dong-A University College of Medicine, Busan 49201, Republic of Korea; 2 Department of Anatomy and Acupoint, College of Korean Medicine, Gachon University, Seongnam 13120, Republic of Korea; anatomy@gachon.ac.kr; 3 Department of Anatomy, The Soonchunhyang University College of Medicine, Cheonan 31151, Republic of Korea

**Keywords:** popliteus muscle, posterolateral complex, neuromuscular compartmentalization, intramuscular innervation, Sihler’s staining, ultrasonography

## Abstract

**Background/Objectives:** The popliteus muscle (PM) plays a crucial role in stabilizing the posterolateral aspect of the knee. However, its layered structure and innervation are not well understood due to its location, size, and proximity to neighboring anatomical features. This study aimed to clarify the layered morphology, intramuscular innervation, and fiber-type composition of the PM, providing anatomical insights for clinical interventions. **Methods:** We examined 32 lower extremities from sixteen formalin-embalmed cadavers using a multimodal approach that included gross dissection, Sihler’s staining, ultrasonography, and histochemical analysis. **Results:** On average, 2.8 ± 1.1 branches of the tibial nerve entered the PM, with a consistently high-density entry zone located at 56–64% of the muscle length. Sihler’s staining and ultrasonographic analyses revealed a distinct separation between the superficial and deep layers across the central tendon, each exhibiting compartmentalized intramuscular branching territories. The superficial layer was primarily composed of type IIx fibers and exhibited a larger pennation angle, while the deep layer was richer in type IIA fibers with a smaller pennation angle. These findings illustrate that the PM functions as a dual motor unit rather than a uniform structure. **Conclusions:** The PM exhibits a distinct compartmentalized organization, functioning as a multifunctional motor unit. The identification of specific intramuscular entry zones and the organization of muscle layers provide strong anatomical evidence for improved targeting in neuromuscular-modulating interventions. This enhances the precision, safety, and efficacy of clinical strategies aimed at addressing posterior knee stability and pathologies related to the posterolateral complex (PLC).

## 1. Introduction

The popliteus muscle (PM) is essential for initiating knee flexion by unlocking the joint and regulating internal tibial rotation, significantly enhancing the posterolateral stability of the knee [1,2,3]. Anatomically, the PM originates from the lateral femoral condyle and the posterior horn of the lateral meniscus, inserting onto the posterior surface of the tibia above the soleal line [4,5,6]. It forms a dynamic tendon–muscle complex through its connections with the joint capsule and the popliteofibular ligament, which contributes to the active stabilization of the posterolateral complex (PLC) [7,8]. Recent studies have redefined the PM as more than just a mechanical structure; it plays a vital role in sensory-motor integration, particularly regarding proprioception [9,10,11]. Dysfunction of the PM can lead to impaired proprioception, altered gait patterns, and chronic posterolateral instability, often presenting as nonspecific lateral knee pain [10,12,13,14,15].

The anatomical complexity of the popliteal region, particularly the close proximity of the tibial nerve, popliteal vessels, and surrounding fascial layers, makes it vulnerable to neural entrapment, especially in cases of chronic mechanical stress, fibrotic changes, or localized inflammation [16,17,18]. Although the functional importance of the PM is well established, its deep anatomical position, compact muscle volume, and symptomatic overlap with adjacent structures—such as the lateral meniscus, joint capsule, and the lateral head of the gastrocnemius—often lead to delayed diagnoses or clinical under-recognition of PM-related disorders [5,18,19].

Comprehensive anatomical data are vital for enhancing the safety and effectiveness of advanced interventions like botulinum toxin (BoNT) injections, ultrasound-guided nerve blocks, and arthroscopy-assisted PLC reconstruction [20,21]. Specifically, the success of BoNT injections hinges on the accurate localization of neural entry points and motor endplate zones, enabling targeted paralysis while maintaining functional capacity [22,23].

Previous anatomical studies have described the popliteus muscle mainly in terms of its origin–insertion anatomy, tendon morphology, and biomechanical role in posterolateral knee stability. Some reports have noted variations in fascicular orientation and tendon expansion patterns, suggesting internal structural complexity. Other investigations have focused on the general course of the tibial nerve [24,25,26,27] and the mechanical relationships between the popliteus muscle, ligaments, and joint capsule [28,29]. However, these studies primarily addressed macroscopic or extrinsic features rather than the intrinsic architecture of the muscle belly. Detailed analyses of intramuscular compartmentalization, layer-specific neural entry patterns, and fiber-type distribution remain limited. To date, no study has integrated gross morphology, intramuscular nerve mapping, and histochemical fiber composition within a single architectural framework.

Therefore, this study aims to use an integrated approach that combines morphometric analysis, Sihler’s nerve staining, immunohistochemical fiber typing, and high-resolution ultrasonography to thoroughly analyze the anatomical and histological features of the PM. Understanding these structural characteristics and neural architecture will provide a solid foundation for developing neuro-muscular-targeted intervention strategies. These insights are expected to improve the precision, safety, and clinical effectiveness of targeted treatments and reconstructive procedures related to the posterior knee joint, while also enhancing pain management and functional rehabilitation.

## 2. Materials and Methods

The experimental procedures in this study received approval from the Institutional Review Board at Soonchunhyang University College of Medicine (SCH IRB approval number: 2025-05-016-002, approval date 17 July 2025). All cadaveric specimens were sourced from the official body donation program of Soonchunhyang University, with each donor having provided written, documented consent for the use of their bodies in medical education and research prior to their death. The authors affirm that every effort was made to adhere to all relevant local and international ethical guidelines and laws governing the use of human cadaveric donors in anatomical research [30].

### 2.1. Dissection and Morphological Analysis of the Popliteus Muscle

This study included 32 lower extremities from 16 formalin-embalmed cadavers (7 males and 9 females; mean age 87.9 years, range 80–95 years) with no documented lower limb pathology or surgical history. After removing the skin and subcutaneous tissue in the popliteal fossa, the gastrocnemius and soleus muscles were carefully dissected to reveal the PM and the tibial nerve.

**Figure 1:**
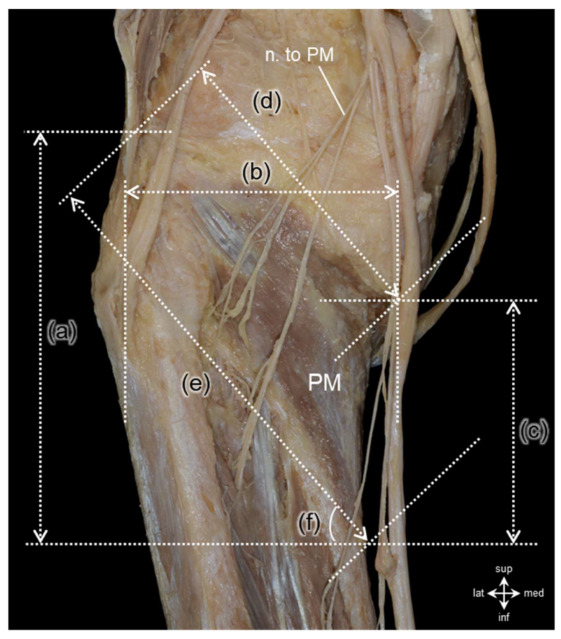
Morphometric parameters of the popliteus muscle (PM). (a) vertical length of the PM. (b) horizontal length of the PM. (c) length of the insertion of the PM. (d) superior border of the PM. (e) inferior border of the PM. (f) angle of the PM. This figure demonstrates the anatomical boundaries and measured parameters of the popliteus muscle on the posterior aspect of the knee. The measured dimensions include: (a) vertical length of the PM (mean: 104.78 ± 13.20 mm), (b) horizontal length (51.38 ± 4.13 mm), (c) length of tibial insertion (65.98 ± 11.15 mm), (d) length of the superior border (66.82 ± 8.19 mm), and (e) length of the inferior border (111.13 ± 11.09 mm). (f) The angle formed between the superior and inferior borders of the muscle was 64.53 ± 5.11°.

We quantitatively measured the morphological characteristics of the PM, including vertical length, horizontal width, and angular orientation, using a digital caliper (NTD15P-15C, Mitutoyo, Kanagawa, Japan) and a digital goniometer (Base-line^®^ Digital Absolute Axis™ Goniometer 12-1027, Fabrication Enterprises Inc., New York, NY, USA) (Figure 1). The nerve to the PM, a branch of the tibial nerve, was meticulously traced from its origin to the point of intramuscular entry at the inferior border of the PM. To establish a standardized reference system, we defined a two-dimensional Cartesian coordinate grid with the most prominent point of the fibular head as the origin (0, 0). We recorded the number of intramuscular nerve branches along with their respective horizontal (*X*-axis) and vertical (*Y*-axis) coordinates. All dissections and measurements adhered to standardized anatomical landmarks. To ensure measurement reliability and minimize inter-observer variability, two anatomists independently conducted all evaluations, and the average values were used for analysis.

### 2.2. Modified Sihler’s Staining

After completing morphometric measurements and nerve mapping, each PM was carefully detached from its origin and insertion, ensuring the nerve integrity to the PM was preserved. A total of 26 PMs underwent Sihler’s whole-mount nerve staining to visualize the intramuscular nerve arborization, following established protocols [31,32].

Initially, the PMs were thoroughly rinsed under running tap water and fixed in a 10% un-neutralized formalin solution for one week. To facilitate maceration and depigmentation, the fixed muscles were immersed in a 3% aqueous potassium hydroxide (KOH) solution containing 0.01% hydrogen peroxide (H_2_O_2_) for three weeks, aiding in tissue softening and pigment removal. Afterward, the tissues were washed again under running water and transferred to Sihler I solution (a mixture of glacial acetic acid, glycerin, and distilled water in a 1:1:6 ratio) for two weeks to achieve decalcification and whitening.

Next, the entire muscle, including the nerve branches, was stained using Sihler II solution (Ehrlich’s hematoxylin, glycerin, and distilled water in a 1:1:6 ratio) for three days. Following the staining process, the samples were returned to Sihler I solution for three hours to selectively remove excess dye from non-neural tissues. To neutralize the acid and enhance nerve contrast, the samples were immersed in a 0.05% lithium carbonate solution for one hour. Finally, for tissue clearing, the muscles were sequentially transferred through graded glycerin concentrations (70%, 80%, 90%, and 100%), with each step lasting 24 h, to render the specimens translucent and suitable for macroscopic evaluation of nerve pathways within the muscle.

For the quantitative analysis of nerve distribution, each stained PM was divided into a 10 × 5 grid, creating 50 regions. The vertical axis (superior-to-inferior) was divided into 10 equal segments, while the horizontal axis (medial-to-lateral, from origin to insertion) was divided into 5 segments. The density and distribution of nerve branches were then assessed within each grid cell.

### 2.3. Ultrasound Examination

The structural characteristics and pennation angles of the PM were assessed using real-time two-dimensional B-mode ultrasound (HS50; Samsung Medison, Seoul, Republic of Korea) with a 40-mm linear array transducer (LA3-14AD; 3–14 MHz, Samsung, Seoul, Republic of Korea). The study involved four fresh cadavers (two males and two females), examining a total of eight lower limb sides. Ultrasonographic assessments were performed prior to formalin injection on specimens distinct from the formalin-fixed cohort used for anatomical and histological analyses. Each measurement was repeated ten times and averaged. Although formal inter-rater reliability statistics were not calculated, pennation angle measurements were refined through repeated calibration to enhance consistency. Each cadaver was positioned prone, with the knee flexed at approximately 20° and the ankle joint in a neutral position. The ultrasound penetration depth was adjusted between 4 and 7 cm, depending on body size. The transducer was aligned perpendicularly to the skin surface, and acoustic gel was applied to ensure high-resolution images. Measurement sites on the skin were marked with a surgical pen to maintain consistency across repeated scans. A virtual transverse line was drawn from the proximal origin to the distal insertion of the PM, serving as a reference for probe placement. The transducer was initially positioned longitudinally, then moved medially and laterally to identify local anatomical structures. Finally, it was rotated to an oblique orientation to observe the fascicle arrangement in the central region of the muscle. Ultrasound imaging revealed distinct divisions of the PM into three layers: the superficial layer, central tendon, and deep layer. The pennation angle was defined as the angle between the muscle fascicles and the long axis of the central tendon. Fascicle angles were measured for both the superficial and deep layers. Each measurement was performed three times, and the mean values were calculated. Statistical analyses were conducted using Microsoft Excel (Microsoft Corp., Redmond, WA, USA), and all ultrasound images were analyzed with ImageJ 1.54 software. Results are presented as mean ± standard deviation, with comparisons made between different regions and layers.

### 2.4. Histological Analysis: ATPase Staining

Superficial and deep tissue samples of human PM were embedded in optimal cutting temperature compound, rapidly frozen in isopentane cooled by liquid nitrogen, and sectioned to a thickness of 8–10 μm using a cryostat set at −20 °C. The sections were then mounted on glass slides, air-dried, and either processed immediately or stored at –80 °C for later analysis. Fiber-type analysis was conducted using myofibrillar ATPase histochemistry, following the method outlined by Guth and Samaha [33]. Serial sections were preincubated in buffer solutions with specific pH levels (4.3, 4.6, and 10.4) to assess ATPase activity under acidic and alkaline conditions. They were then incubated in an ATP substrate solution and treated sequentially with calcium, cobalt, and ammonium sulfide to visualize the reaction. Based on staining intensity at each pH level, fibers were classified as Type I (slow oxidative) or Type II (fast-twitch), with Type I fibers appearing dark and Type II fibers lighter. Subclassification of Type II fibers into Type IIa and Type IIx was determined based on classical myofibrillar ATPase pH sensitivity patterns according to established histochemical criteria, with differential staining profiles observed across serial sections preincubated at pH 4.3, 4.6, and 10.4. Fiber classification was based on classical myofibrillar ATPase pH sensitivity patterns according to established histochemical criteria. Type IIa fibers exhibited moderate staining following acidic preincubation and strong reactivity at alkaline pH (10.4), whereas Type IIx fibers demonstrated reduced staining under acidic conditions and comparatively stronger alkaline reactivity. Classification was determined by matching individual fibers across serial sections and evaluating their relative staining patterns rather than relying on single-section intensity differences, in accordance with established histochemical criteria. Images were captured using a BX43 microscope (Olympus, Tokyo, Japan), and three random fields from each sample were analyzed. Fiber classification was conducted manually by two independent, blinded observers by matching corresponding fibers across serial sections and confirming consistent staining profiles at each pH condition. Staining intensity and fiber cross-sectional area were quantified in pixels using ImageJ software (NIH, Bethesda, MD, USA). The quantitative data are expressed as mean ± standard deviation. Differences in fiber-type proportions between superficial and deep layers were analyzed using independent *t*-tests. Inter-observer reliability was assessed using the intraclass correlation coefficient. All statistical analyses were performed using Microsoft Excel (Microsoft Corp., Redmond, WA, USA), with statistical significance set at *p* < 0.05.

## 3. Results

### 3.1. Morphological Characteristics of the Popliteus Muscle

The average height of the PM was 104.8 ± 13.2 mm, and its average width was 51.4 ± 4.1 mm. The lengths of the superior and inferior borders were 66.8 ± 11.2 mm and 111.1 ± 11.1 mm, respectively. The overall orientation angle of the muscle was measured at 64.5 ± 5.1°, indicating a fan-shaped morphology. No significant differences were observed between the left and right sides for any of the morphometric parameters. In contrast, significant differences were observed only by sex for horizontal length, superior border, and inferior border. All of which were longer in males than in females (*p* < 0.05) (Figure 1, Table 1).

### 3.2. Innervation of the Popliteus Muscle

In all specimens (*n* = 32), the PM was innervated by branches of the tibial nerve, specifically the nerve to the PM, which originated in the popliteal fossa and traveled distally along the muscle surface before entering through the inferior border. On average, there were 2.8 ± 1.1 nerve branches per specimen. A single branch was present in all cases (100.0%, 32/32), while two branches were observed in 56.3% (18/32) and three branches in 18.8% (6/32) of the specimens.

**Figure 2:**
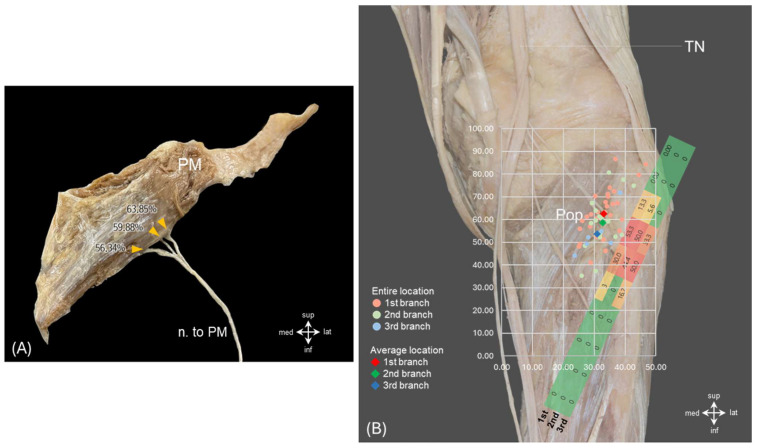
Anatomical locations and branching patterns of tibial nerve entries to the popliteus muscle. (**A**) Macroscopic dissection of the popliteus muscle and its innervating branches from the tibial nerve. The first branch (1st) was positioned most proximally and laterally, entering near the origin of the muscle. Subsequent branches (2nd and 3rd) entered more distally and medially, suggesting a layered nerve entry pattern. (**B**) Grid-based mapping of nerve entry points to the popliteus on the posterior knee. Each circle represents an individual entry point, color-coded by branch order (red: 1st, green: 2nd, blue: 3rd), while solid diamonds (◆) indicate mean coordinates for each branch. The distribution pattern demonstrates a consistent and clustered entry zone, indicating a stereotypical spatial relationship between the tibial nerve and the popliteus muscle.

To standardize the measurements of entry points, the muscle insertion was defined as 0% and the origin as 100%. Using this reference, the average neural entry location was found to be 61.7 ± 6.9% from the insertion. The branches were sequentially labeled as the 1st, 2nd, and 3rd based on their proximity to the muscle origin. The 1st branch entered at 63.9 ± 6.7%, typically in a superolateral direction; the 2nd branch at 59.9 ± 5.8%; and the 3rd branch at 56.3 ± 7.8%, commonly in an inferomedial direction (Figure 2A, Table 2). The high-density neural entry zone was consistently located in the posterior mid-portion of the muscle, specifically in the superolateral one-third region (56–64% from the insertion). This zone displayed a clustered pattern on the grid-based heatmap analysis (Figure 2B, Table 2).

### 3.3. Intramuscular Nerve Distribution (Modified Sihler’s Staining)

**Figure 3:**
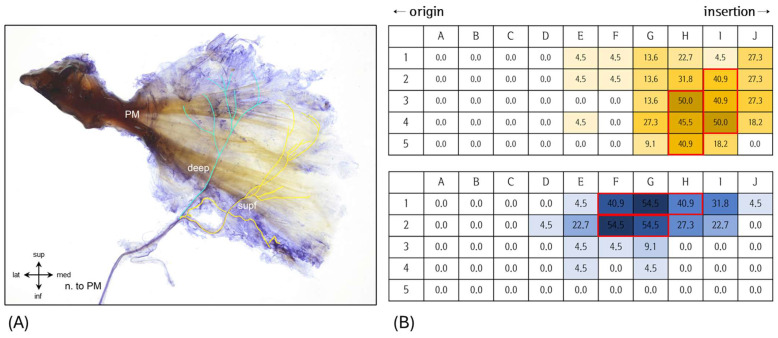
Intramuscular distribution of tibial nerve branches to the popliteus muscle visualized via Sihler’s staining. (**A**) Posterior view of the popliteus muscle showing intramuscular nerve arborization patterns divided into a 10 × 5 grid from the lateral-superior (origin) to the medial-inferior (insertion) direction. The muscle was distinctly divided into superficial and deep layers based on the central tendon. (**B**) Heatmaps demonstrate the proportional neural entry density across grid zones. Superficial branches predominantly arborized in the inferolateral direction (3–5 o’clock), with the highest densities observed in H3, I4 (50.0%), H4 (45.5%), and H5, I2–3 (40.9%). Deep branches showed a clustered superomedial distribution (11–1 o’clock), with maximal innervation at F2 and G1–2 (54.5%), followed by F1 and H1 (40.9%). Overlapping zones between superficial and deep branches were most frequent at H2, followed by H1 and I1. Color-coded boxes indicate relative innervation density (yellow/red box for superficial, blue/red box for deep), with darker shades representing higher density.

Sihler’s whole-mount staining revealed a clear bipartite organization of the PM, consisting of distinct superficial and deep layers separated by the central tendon (Figure 3A,B). The patterns of nerve arborization varied significantly between these compartments. Superficial branches were widely distributed around the insertion area, extending primarily in an inferolateral direction (3–5 o’clock). Grid-based density mapping indicated the highest frequencies in regions H3 and I4 (50.0%), followed by H4 (45.5%), H5 (40.9%), and I2–3 (40.9%) (Figure 3B). In contrast, deep branches were mainly concentrated near the origin, particularly around the proximal central tendon, and projected in a superomedial direction (11–1 o’clock). The highest densities in this layer were found in regions F2 and G1–2 (54.5%), followed by F1 and H1 (40.9%) (Figure 3B). The overlapping areas common to both layers were most pronounced in H2, followed by H1 and I1, indicating co-localized nerve entry across fascial planes.

### 3.4. Topographic Relationship with Nerve to Tibialis Posterior

**Figure 4:**
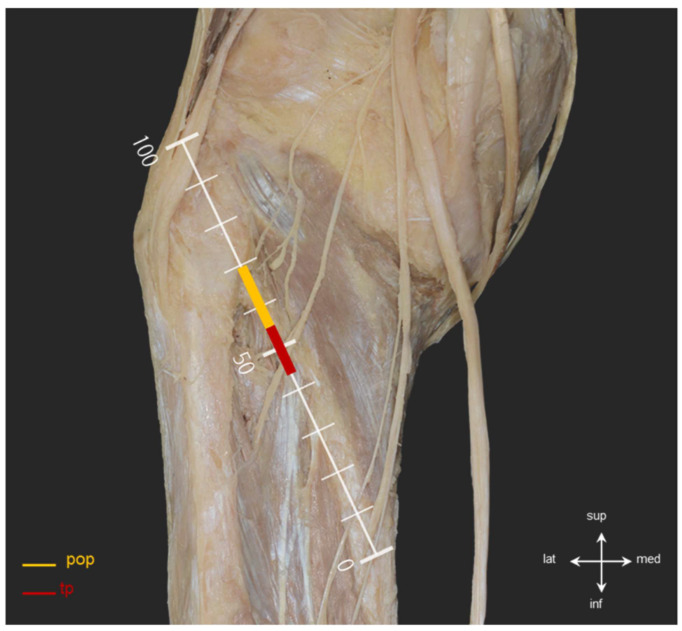
Topographic course of nerves to the popliteus and tibialis posterior muscles and identification of the neurovascular danger zone. This image illustrates the relative anatomical positions of the nerve to the popliteus (pop, yellow) and the nerve to the tibialis posterior (tp, red) on the posterior aspect of the knee. The vertical reference axis (0–100%) was established from the most prominent point of the fibular head, designated as the origin (100%). The red-highlighted segment (average 51.3% region) marks a potential danger zone where the tp nerve overlaps with common injection fields targeting the popliteus muscle. Awareness of this spatial relationship is crucial to avoid iatrogenic injury during procedures such as botulinum toxin injection or posterior arthroscopic approaches.

Two distinct nerves were identified on the surface of the PM: the *n*. to PM and the *n*. to TP. Using the most prominent point of the fibular head as the reference origin (0, 0), we measured the coordinates of the nerve entry points along the X (horizontal) and Y (vertical) axes. The average coordinate values for the *n*. to PM branches were as follows: 1st branch: 19.1 ± 4.8 (X), 28.5 ± 1.6 (Y), 2nd branch: 20.8 ± 4.4 (X), 35.7 ± 3.8 (Y), 3rd branch: 23.2 ± 4.5 (X), 37.1 ± 4.8 (Y). The *n*. to TP was located more inferomedially, at 25.8 ± 2.6 (X), 41.6 ± 5.2 (Y) (Figure 2B, Table 2). Thus, the 1st branch of the *n*. to PM was positioned most superolaterally relative to the fibular head (highlighted in yellow), while the *n*. to TP was situated in the inferomedial region, approximately 51.3% from the insertion (highlighted in red) (Figure 4, Table 2).

### 3.5. Ultrasound Scanning Analysis

**Figure 5:**
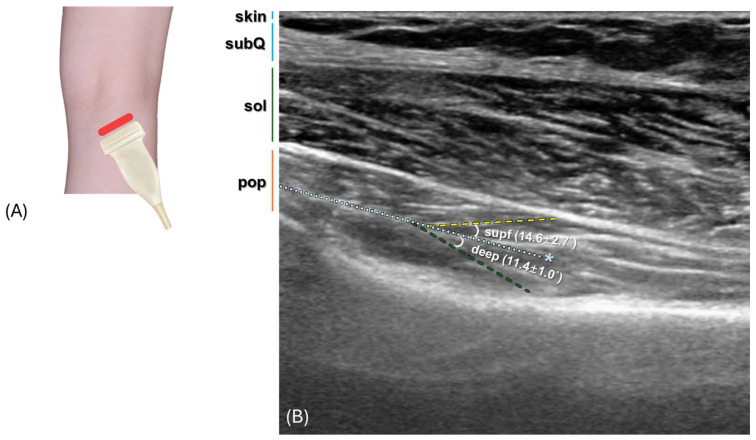
Ultrasound image of the popliteus muscle. (**A**) Probe positioning for oblique scanning at three standardized locations on the popliteal fossa. (**B**) Ultrasound image on B site. The popliteus muscle was divided into a superficial (yellow line) and a deep (green line) layer based on the central tendon (asterisk). subQ, subcutaneous tissue, sol, soleus muscle, pop, popliteus muscle.

High-resolution ultrasound analysis was conducted on eight cadaveric lower limbs, revealing consistent findings across all specimens. The PM was clearly identified in the central region (Figure 5A), with the central tendon most distinctly visualized in the middle area. This landmark allowed for a clear separation of the muscle into a superficial layer and a deep layer. Imaging showed that the superficial layer was relatively thin, with a well-defined border adjacent to the central tendon, while the deep layer exhibited a more homogeneous echogenic pattern. Notable differences in fascicle orientation and radial architecture were also observed between the two layers. Quantitative measurements indicated that the superficial layer had a mean pennation angle of 14.6° ± 2.7°, compared to the deep layer’s smaller mean angle of 11.4° ± 1.0° (Figure 5B).

### 3.6. Histological Analysis

**Figure 6:**
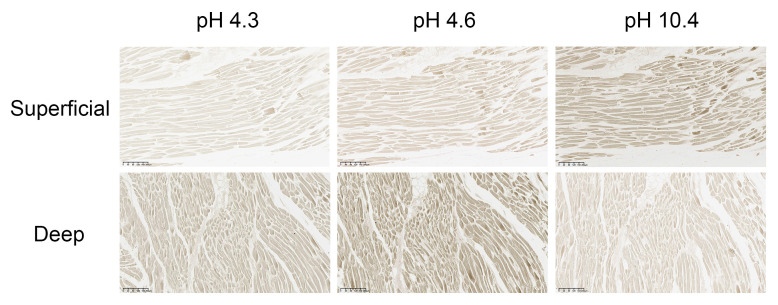
ATPase staining characteristics of superficial (Type IIx) and deep (Type IIa) muscle fibers under different pH conditions. The indicated pH values correspond to the pre-incubation conditions used in myofibrillar ATPase histochemistry for muscle fiber type differentiation. Representative ATPase-stained cross-sections of superficial (Type IIx-dominant) and deep (Type IIa-dominant) muscle layers at pH 4.3, 4.6, and 10.4 (*n* = 6). Mean staining intensities (arbitrary units) are indicated below each image. Superficial fibers exhibited markedly stronger staining at pH 10.4 (219.8 ± 0.2) compared to pH 4.6 (174.1 ± 0.1) and pH 4.3 (149.8 ± 0.1), consistent with the characteristics of Type IIx fast glycolytic fibers. In contrast, deep fibers showed moderate and relatively stable intensities across pH values (194.7–201.5), which corresponds to Type IIa fast oxidative glycolytic fibers. At pH 4.3, deep fibers stained significantly more intensely than superficial fibers (*p* < 0.05), indicating greater oxidative endurance capacity in the deep compartment. Scale bars = 100 μm.

ATPase histochemistry (*n* = 6) revealed distinct differences in fiber-type composition between the superficial and deep layers (Figure 6). In the superficial layer, staining intensity significantly increased under alkaline conditions (pH 10.4: mean = 219.8 ± 0.2%, area = 28.6%) compared to acidic conditions (pH 4.6: mean = 174.1 ± 0.1%, area = 14.3%). Strongly acidic conditions (pH 4.3) yielded the lowest intensity (149.8 ± 0.1%). This pronounced response to alkaline conditions is characteristic of Type IIx fast glycolytic fibers, indicating a predominance of fibers adapted for rapid contraction and glycolytic metabolism in the superficial compartment.

In contrast, fibers in the deep layer exhibited only modest variations across different pH conditions (pH 4.3: 194.7 ± 0.2%; pH 4.6: 201.5 ± 0.1%; pH 10.4: 185.8 ± 0.3%) and comprised 13–15% of the total area. This stable staining pattern corresponds to Type IIA fast oxidative glycolytic fibers, suggesting that the deep compartment is optimized for higher oxidative capacity and fatigue resistance compared to the superficial layer. Notably, under pH 4.3 conditions, which selectively stain Type I fibers, the deep layer (194.7 ± 0.2%) demonstrated a significantly greater proportion than the superficial layer (149.8 ± 0.1%; *p* < 0.05), confirming that the deep compartment possesses enhanced oxidative endurance characteristics.

## 4. Discussion

This study reveals that the PM is not a morphologically uniform structure; rather, it is a bi-layered muscle with distinct functional compartments and a highly organized neuromuscular architecture. By combining gross anatomical dissection, Sihler’s nerve staining, high-resolution ultrasonography, and histochemical fiber-type analysis, we present converging evidence that the superficial and deep layers of the PM are separate neuromuscular compartments divided by the central tendon. Each layer exhibits a unique pattern of intramuscular innervation and muscle fiber composition.

From a purely anatomical standpoint, the concordance between the central tendinous partition, layer-specific pennation patterns, and segregated intramuscular nerve territories indicates that the PM is organized as a structurally compartmentalized entity. The central tendon appears not merely as an internal aponeurotic support, but as a morphological boundary that aligns with distinct neuromuscular domains. Such correspondence between architectural planes and neural distribution suggests that the observed bilayered configuration reflects a consistent intrinsic structural organization rather than a random architectural variation. This structural integration reinforces the concept that intramuscular organization within the PM follows a defined compartmental blueprint.

On average, 2.8 ± 1.1 tibial nerve branches entered the PM, with a consistently high-density entry zone located at approximately 56–64% of the muscle length along the posterolateral margin near the fibular head (Figure 2, Figure 3 and Figure 4, Table 1 and Table 2). Grid-based Sihler’s staining revealed that these branches did not arborize diffusely throughout the muscle; instead, they established layer-specific intramuscular territories separated by the central tendon (Figure 3). This spatial organization suggests that motor unit recruitment and force modulation within the PM may be regulated in a layer-dependent manner, rather than through uniform activation of the entire muscle [10,34,35,36].

Histochemical and architectural findings further support this interpretation. The superficial layer, located inferolaterally (approximately 3–5 o’clock), was primarily composed of type IIx fibers, while the deep layer, situated superomedially (approximately 11–1 o’clock), exhibited a predominance of type IIA fibers (Figure 6). Although ATPase histochemistry remains a classical and widely used method for fiber-type differentiation, it may not fully resolve hybrid or transitional fibers without complementary immunohistochemical confirmation. Therefore, some degree of overlap between Type IIa and Type IIx fibers cannot be entirely excluded. When considered alongside the differences in pennation angle (Figure 5B), these results suggest distinct mechanical roles for each layer [37]. The superficial layer had a relatively larger pennation angle, a characteristic typically associated with increased fiber packing and enhanced force-generating capacity [38,39,40]. This configuration aligns with the demands for dynamic stabilization, particularly in resisting loading and initiating internal tibial rotation during early knee flexion [2,41]. In contrast, the deep layer of the muscle exhibits a smaller pennation angle, with fibers oriented more parallel to the joint’s axis of rotation. Its continuity with the posterior cruciate ligament and the joint capsule allows for sustained tension, precise rotational control, and efficient load distribution across a broad range of knee flexion angles [2,10,42,43,44,45]. These findings challenge the traditional view of the PM as merely an auxiliary internal rotator, instead supporting its role as a composite muscle that provides both phasic (superficial) and tonic (deep) stabilization [2,4,7,10,29,46,47,48,49,50,51]. This compartmentalized model also has significant implications for the function of the posterolateral corner (PLC). The PM serves as the floor of the popliteal fossa and plays a crucial role in internal rotational stability and coordination with PLC structures [52,53]. Clinical assessment of injuries remains challenging. For instance, PLC injuries, which are estimated to account for about 16% of all knee injuries, are often underdiagnosed. This is primarily due to the depth and small size of the PM, its close proximity to the lateral meniscus and joint capsule, and the difficulty in distinguishing it from the lateral head of the gastrocnemius [6,18,19,54,55,56,57,58,59]. Although previous studies have focused on the biomechanical relationships between the PM and surrounding structures [19,28,29], there has been limited detailed spatial mapping of intramuscular innervation and layer-specific functional compartmentalization. Additionally, functional changes in the PM have been observed in conditions like cerebral palsy, hip dysplasia, and pediatric gait disorders [12,60], highlighting the need for a more precise anatomical framework.

The identification of reproducible, layer-specific intramuscular nerve entry zones offers a practical anatomical foundation for image-guided evaluation and intervention. The effectiveness of botulinum toxin (BoNT) relies heavily on precisely targeting these neural entry regions [20,21], making the definition of consistent entry patterns clinically significant. BoNT works by inhibiting acetylcholine release at the neuromuscular junction and is used to reduce muscle tone and alleviate pain in various neuromuscular conditions [22,23]. In this study, tibial nerve branches were found to consistently cluster along the posterolateral fascial margin, displaying specific convergence patterns across different layers (Figure 2, Figure 3 and Figure 4, Table 2). These findings suggest anatomically consistent target zones that may improve precision in ultrasound-guided BoNT injection, selective rehabilitation strategies, and posterior arthroscopic procedures [50,61,62,63,64,65]. Importantly, the identified convergence regions (H2, H1, I1) may serve as practical injection sites that facilitate efficient targeting while minimizing invasiveness (Figure 4, Table 2). In contrast to the relatively stable convergence zones along the posterolateral fascial margin, the tibialis posterior–related branch located at approximately 51.3% of muscle length demonstrated greater positional variability and closer proximity to adjacent posterior compartment structures. This region therefore warrants procedural caution, as variability in branch location may influence optimal injection depth and needle trajectory during image-guided interventions [46]. Precise localization of intramuscular neural entry zones is particularly relevant in botulinum toxin applications, where therapeutic efficacy depends on accurate targeting of motor endplate–rich regions. Furthermore, given the anatomical complexity of the posterolateral corner and the known risk of tibial nerve injury in posterior knee approaches, recognition of this variable region may assist in safer portal placement and trajectory planning during arthroscopic or open procedures [66,67,68].

Several limitations must be acknowledged. Formalin-fixed cadaveric specimens do not accurately replicate in vivo muscle tone, elasticity, or dynamic neuromuscular behavior. The advanced mean age of the cadaveric cohort (87.9 years) represents an additional important consideration. None of the specimens demonstrated gross focal muscle atrophy, prior surgical alteration, or overt structural distortion upon dissection. However, age-related sarcopenic changes, including potential shifts in fiber-type composition or motor unit remodeling, cannot be entirely excluded. While such changes may influence quantitative fiber proportions, the consistent bilayered architectural organization and reproducible intramuscular nerve entry patterns observed across specimens suggest that the identified compartmental structure is unlikely to be solely attributable to age-related degeneration. Furthermore, the relatively small sample size, which mainly includes elderly donors, restricts the generalizability of the findings across different age groups and sexes. Additionally, Sihler’s staining does not differentiate between motor and sensory fascicles, potentially underestimating terminal arborization. Future research should incorporate immunohistochemical techniques for more detailed characterization, mapping of intrafusal elements, and electromyography-based dynamic assessments to further validate functional interpretations and enhance clinical applications [69]. Despite these limitations, the current study integrates ultrasonography, nerve mapping, and fiber-type analysis, establishing a solid anatomical foundation for understanding the PM as a bilayered, functionally specialized muscle. This work also identifies clinically actionable targets relevant to PLC-related knee pathology [52,53,54].

## 5. Conclusions

The PM is organized into distinct layers. The superficial layer, oriented inferolaterally and composed of type IIx fibers with a larger pennation angle, is specialized for dynamic stabilization. In contrast, the deep layer, oriented superomedially and made up of type IIA fibers with a smaller pennation angle, serves as a module for static stabilization and proprioceptive regulation. This layered structure positions the PM as a multifunctional motor unit rather than merely an auxiliary rotator. A key finding of this study is that the consistently identified intramuscular entry zones and their branching patterns provide strong anatomical evidence for precise targeting in procedures such as botulinum toxin injection, ultrasound-guided nerve block, and PLC reconstruction, thereby improving both accuracy and safety.

## Figures and Tables

**Table 1:** Morphometric parameters of the popliteus muscle by sex and laterality. Sex- and side-based comparisons were conducted for all morphometric parameters, and corresponding *p*-values have been added to this table. No statistically side-related significant differences were observed.

	Sex			Side			Total (*n* = 32)
	Male (*n* = 14)	Female (*n* = 18)	*p*-Value	Left (*n* = 16)	Right (*n* = 16)	*p*-Value	
Vertical length (a)	109.63 ± 11.32	102.36 ± 13.66	0.16	105.78 ± 14.25	103.92 ± 12.61	0.57	104.78 ± 13.20
Horizontal length (b)	54.60 ± 4.64	49.76 ± 2.75	0.01	50.37 ± 3.90	52.26 ± 4.24	0.27	51.38 ± 4.13
Length of insertion (c)	68.66 ± 6.18	64.37 ± 13.17	0.22	64.43 ± 10.57	67.34 ± 11.79	0.42	65.98 ± 11.15
Superior border (d)	72.66 ± 9.29	63.31 ± 5.01	0.00	66.94 ± 9.59	66.70 ± 7.04	0.89	66.82 ± 8.19
Inferior border (e)	117.68 ± 10.50	107.20 ± 9.67	0.00	113.54 ± 13.32	109.01 ± 8.53	0.12	111.13 ± 11.09
Angle (f)	63.82 ± 5.77	64.96 ± 4.78	0.54	63.71 ± 3.80	65.26 ± 6.07	0.19	64.53 ± 5.11

**Table 2:** Coordinates and relative positions of nerves around the popliteus muscle.

	*X*-Axis (mm)	*Y*-Axis (mm)	Location (%)
*n*. to pop			
1st	19.1 ± 4.8	28.5 ± 1.6	63.9 ± 6.7
2nd	20.8 ± 4.4	35.7 ± 3.8	59.9 ± 5.8
3rd	23.2 ± 4.5	37.1 ± 4.8	56.3 ± 7.8
mean	20.4 ± 4.4	32.7 ± 4.9	61.7 ± 6.9
*n*. to tp	25.8 ± 2.6	41.6 ± 5.2	51.3 ± 3.8

## Data Availability

The data presented in this study are available from the corresponding author upon reasonable request, as the cause of death and relevant pathology are considered personal data.

## Abbreviations

The following abbreviations are used in this manuscript:

BoNT	Botulinum toxin
PCL	Posterior cruciate ligament
PLC	Posterolateral complex
PM	Popliteus muscle
TP	Tibialis posterior
